# Adherence to the Mediterranean diet, kinanthropometric characteristics and physical performance of young male handball players

**DOI:** 10.7717/peerj.14329

**Published:** 2022-12-01

**Authors:** David Romero-García, Francisco Esparza-Ros, María Picó García, José Miguel Martínez-Sanz, Raquel Vaquero-Cristóbal

**Affiliations:** 1 Nursing Department, Food and Nutrition Research Group (ALINUT), Faculty of Health Sciences, University de Alicante, San Vicente del Raspeig, Alicante, Spain; 2 Research Group on Prevention of Sports Injuries, International Chair of Kinanthropometry, Catholic University of Murcia, Murcia, Spain; 3 Research Group on Prevention of Sports Injuries, Faculty of Sport, Catholic University of Murcia, Murcia, Spain

**Keywords:** Handball, Body composition, Mediterranean diet, Sports performance

## Abstract

**Introduction:**

Handball is a team sport whose performance depends on a large number of factors. The objectives of the present study were to analyze the differences in physical performance, kinanthropometric variables, and adherence to the Mediterranean diet (MD), in handball players according to sports category, as well as the possible relationships between these variables.

**Methods:**

One hundred and thirty-three male handball players (35 infant; 46 cadets; 26 juniors and 26 seniors players) underwent a kinanthropometric analysis following the ISAK protocol, self-completed the KIDMED questionnaire, and their physical condition was evaluated with different physical tests.

**Results:**

Significant differences (*p* < 0.001–0.007) were found between the sports categories in most of the physical condition variables and anthropometric characteristics, but not in the degree of adherence to the MD. The predominant level of adherence to the MD was medium (47.4%), followed by good (42.1%), and correlated with the body mass, the height, the BMI, the muscle mass and the ∑3-girths sum corrected, but not with physical test results. A multiple linear regression analysis showed that the fat mass and muscle mass variables had a more specific weight in the results of the physical fitness tests.

**Conclusions:**

There are differences according to sports category in kinanthropometric characteristics and physical fitness tests in adolescent handball players. The predominant degrees of adherence to the MD were medium and good. A relationship was found between anthropometric characteristics and physical performance in handball players.

## Introduction

Handball is a complex, multifactorial, and high-contact team sport, in which different actions, movements or plays occur intermittently at very high intensity (Martínez-Rodríguez et al., 2020). These periods alternate with brief moments of low intensity that serve as a recovery period before the next play (Hermassi, Laudner & Schwesig, 2019; Hermassi et al., 2020). All these situations are influenced by the physical capabilities of the players themselves highlighting strength, endurance, speed, and flexibility (Hammami et al., 2019; Molina-López et al., 2020).

Considering the characteristics of the game, there is no doubt that handball players need to have a correct physical preparation to be able to overcome all the physical demands and supply the energy required during a training session or match. Such physical preparation is related to the anthropometric characteristics and body composition of the individual players, which influence their performance during competition (Ghobadi et al., 2013; Martínez-Rodríguez et al., 2020). In fact, both the anthropometry and body composition of the players are important factors that determine performance within a team (Matthys et al., 2013).

Generally speaking, players that make up elite teams are tall, with low body fat values, and good muscle development (Vila et al., 2012; Ghobadi et al., 2013). In fact, greater muscle mass also implies improved performance, probably due to increased maximal strength and muscle power (Granados et al., 2015). However, researchers have not yet been able to establish a consensus to be able to exactly define which anthropometric characteristics are more related or have a greater influence on the improvement of performance in handball players.

Another factor that may have a strong influence is the athlete’s diet (Kerksick et al., 2018). In fact, organizations with great international prestige insist that the performance of athletes, as well as their recovery, can be improved with optimal nutrition (Thomas, Erdman & Burke, 2016). With respect to nutrition, it is known that the Mediterranean diet (MD) is a healthy diet model which is characterized by a high consumption of fruits, vegetables, cereals, nuts, legumes and olive oil; a moderately high consumption of fish; a moderate consumption of dairy products; and a low consumption of meat, poultry and foods with saturated fats. A good adherence to MD has been associated with certain physical benefits and high levels of health-related quality of life (Galan-Lopez et al., 2018; Galan-Lopez et al., 2019; Galan-Lopez et al., 2020; Manzano-Carrasco et al., 2020a). This could be because good adherence to this dietary pattern provides a balanced intake of macronutrients such as complex carbohydrates, unsaturated fats and high biological value proteins, as well as micronutrients such as vitamins and minerals (Davis et al., 2015). This adequate intake of macronutrients and micronutrients could favor the energy availability of athletes during competition, promote physiological processes and prevent a positive energy balance that leads to fat accumulation (Tosti, Bertozzi & Fontana, 2018). However, very few studies have analyzed adherence to the MD in young athletes, and how it influences sports performance, body composition characteristics, or both, not finding a high adherence of young athletes to this dietary pattern. (Rubio-Árias et al., 2015; Vaquero-Cristóbal et al., 2018; Manzano-Carrasco et al., 2020b; Martínez-Rodríguez et al., 2021). In addition, no studies have been found about handball players.

Therefore, the objectives of the present study were: (a) to observe whether there is any difference between anthropometric characteristics, sports performance, and adherence to the MD depending on the sports category of handball players; (b) to know the degree of adherence to the MD of the players; (c) to know whether this degree of adherence to the MD influences sports performance and anthropometric characteristics; (d) to know whether there is a relationship between anthropometric characteristics and sports performance. The hypotheses of this study were: (1) athletes of higher categories would show a greater optimization of anthropometric characteristics and better results in the physical condition tests than their equivalent athletes of lower categories; (2) adolescent handball players would have a heterogeneous adherence to DM, with no clear pattern in this regard; (3) a better adherence to DM would positively influence the presence of more optimal anthropometric parameters and sports performance; and (4) adolescent handball players with better body composition, that is, with greater muscle development and a lower adipose component, and with good adherence to DM will have better results in physical fitness tests related to handball performance.

## Materials & Methods

### Study design

The present study was carried out by means of a descriptive-correlational cross-sectional study in which anthropometric characteristics, adherence to the Mediterranean diet, and performance in different physical tests, were evaluated in young handball players. The sample size calculation was performed with Rstudio software (version 3.15.0; RStudio Team, 2018). The significance level was set a priori at *α* = 0.05. The standard deviation (SD) was set according to the muscle mass data from previous studies in adolescent athletes (SD = 2.59) (Albaladejo-Saura et al., 2022). With an estimated error (d) of 0.44 kg, the sample size needed was 133 subjects. The study population was selected through non-probabilistic convenience sampling among clubs with infant, cadet, junior and senior categories in the provinces of Alicante and Murcia (Spain).

### Participants

One hundred and thirty-three male handball players between 12 and 28 years of age voluntarily participated in this study. They were divided according to the category in which they competed, with the final sample being 35 infant (mean age: 13.41 ± 0.40 years), 46 cadets (mean age: 14.83 ± 0.64 years), 26 juniors (mean age: 17.20 ± 0.55 years), and 26 seniors (mean age: 20.09 ± 2.76) years. The inclusion criteria for the study were: (a) at least two years playing handball; (b) being federated in handball; (c) training a minimum of three days per week; and (d) training for at least one month without missing any training. The exclusion criteria for the study were: (a) being injured at the time of the evaluations.

### Procedure

The study was approved by the Ethics Committee of the University of Alicante (code: UA-2022-02-01). In addition, all the players were previously informed about the objective and method of the study, and signed the informed consent before starting the study, or their parents or legal guardians, failing this, in the case of minors.

The measurements were performed in the same week in three evaluation sessions, leaving one rest day between the second and third evaluation day. On the measurement days, the players should not have performed high-intensity exercise the previous day, nor should they have performed training or stretching sessions on the same day. On the first day, kinanthropometric characteristics and adherence to the MD were evaluated; on the second day, agility and endurance tests were performed; and on the last day, jumping, throwing, and sprinting tests were completed, always leaving five minutes of rest between tests.

### Adherence to the Mediterranean diet

To analyze adherence to the MD, participants self-completed the questionnaire that assessed MD quality index in children and adolescents (KIDMED, KM). With a maximum score of 12 points, responses were categorized according to the level of quality of MD: ≤3 poor quality, 4–7 medium quality and ≥8 optimal quality (Serra-Majem et al., 2004).

### Kinanthropometric evaluation

The kinanthropometric evaluation was performed by an anthropometrist level 2 accredited by the International Society for the Advancement of Kinanthropometry (ISAK), with a technical measurement error of 0.03% for basic measurements, 2.24% for skinfolds and 0.36% for girths, and following the international standards recommended by the ISAK (Esparza-Ros, Vaquero-Cristóbal & Marfell-Jones, 2019). The following variables were taken: the four basic measurements (body mass, height, sitting height y arms span), three skinfolds (triceps, thigh and leg), and four girths (relaxed and contracted arm, thigh and leg). For the determination of body mass, a SECA 862 scale (SECA, Hamburg, Germany), with an accuracy of 100 g was used; for height and sitting height, a SECA 217 (SECA, Hamburg, Germany) measuring rod with a one mm accuracy was used; and for wingspan, an Avanutri wingspan meter (Avanutri, Tres Rios, Brazil); for girths, a CESCORF metal tape (CESCORF, Portro Alegre, Brazil) was used; and for skinfolds, a Slimguide plicometer (Creative Health Products, United States) with a 0.5 mm precision was utilized. All the anthropometric measurements were taken two or three times, depending on whether the difference between the first two was greater than 5% for skinfolds, and 1% in the rest of the measurements, taking the mean or median, respectively, for subsequent analyses. The temperature of the room where the measurements were taken was standardized at 24 °C, and all measurements were taken from 15:00 to 21:00.

With the data collected, the following were estimated: body mass index (BMI), with the formula body mass (kg)/height (m)2 (Alvero Cruz et al., 2010); the percentage of fat mass (FM) (Slaughter et al., 1988) and muscle mass (MM) (Poortmans et al., 2005), with the formulas chosen because they have been validated in a growing population (Alvero Cruz et al., 2010); ∑3 skinfolds (triceps, thigh and leg); and ∑3-girths sum corrected (relaxed arm, thigh and leg), with the formula corrected perimeter = perimeter - *π* × fold (Alvero Cruz et al., 2010).

### Physical condition

The T-Half Test protocol (Sassi et al., 2009) was used to evaluate the agility of the players. This test was chosen because unlike the T-Test, it is shorter in distance and more representative for handball. To monitor the start and end of the test, a photocell (Witty, Microgate, Italy) was used.

The endurance capacity of the players was evaluated following the protocol of the Intermittent Recovery Yo-Yo level 1 test (Krustrup et al., 2003). In addition to recording the total meters run by each athlete in this test, the theoretical VO_2_ max was estimated using the equation of Bangsbo, Iaia & Krustrup (2008), which has been previously used in similar contexts (Gonçalves & Carvalho, 2021; Macpherson & Weston, 2015; Köklü et al., 2012).

To assess the jumping ability of the players, they were asked to perform the Squad Jump (SJ) and Counter Movement Jump (CMJ). The latest version of the MyJump application (Balsalobre-Fernández, Glaister & Lockey, 2015) was used to measure the jumps, and a previously described protocol was used (Hermassi et al., 2021).

To assess the players’ upper limb power, they were given the overhead medicine ball throw test, and a tape measure (Haest, Germany) with 0.1 cm accuracy was used to measure the distance (Albaladejo-Saura et al., 2022).

For the assessment of speed, a 30-m sprint was performed at the maximum possible intensity. To monitor the start and end of the test, two photocells (Witty, Microgate, Italy) were used.

The players performed two valid attempts of the agility, jump, throw, and sprint tests, and one attempt of the endurance test. A familiarization with the physical test protocols was performed the week before the measurements. In addition, before sessions 2 and 3, a standardized warm-up was performed consisting of 10 min of continuous running at a gentle jog, dynamic joint mobility exercises and low-intensity exercises, simulating the tests they were going to perform in that session. The researchers encouraged the players to perform the physical tests at the maximum possible intensity.

### Statistical analysis

Both the kinanthropometric characteristics of the players, as well as adherence to the MD and the results obtained in the performance tests, were analyzed using descriptive statistics, to obtain the mean and standard deviation. The Kolmogorov–Smirnov normality test was performed to evaluate that all variables had a normal distribution. The kurtosis was also evaluated, and the Mauchly sphericity test was performed to test the sphericity of the data. Given the normal distribution of the data and to analyze the differences between the categories in the anthropometric variables, adherence to MD, and the results of the physical tests, an analysis of variance (ANOVA) was performed, as well as an analysis of covariance (ANCOVA) to observe the influence of the covariate age. When significant differences were found between groups, a pairwise comparison was performed using the Bonferroni correction for multiple comparisons. In addition, a bivariate correlation was performed between the kinanthropometric characteristics of the players and adherence to MD with the results obtained from the physical tests. The association between these variables was measured as follows: *r* < 0.3, low association; *r* = 0.3–0.5, moderate association; and *r* > 0.5, high association. Finally, a stepwise linear regression analysis was performed on all the variables that were found to correlate with the physical fitness tests. As for the categorical variables, a chi-square test (*χ*2) was performed to analyze the distribution of the degree of adherence to the Mediterranean diet. The minimum level of statistical significance was set at *p* < 0.05. All the data were analyzed using the Statistical Package for the Social Sciences (SPSS) version 25.0 (IBM, Armonk, NY, USA).

## Results

Table 1 shows the descriptive statistics (mean ± standard deviation) of all measured variables, as well as the differences between groups, the main effects of the covariate age and the interaction of the category*age model. When comparing results among categories, significant differences (*F* = 4.259–58.408; *p* < 0.001−0.007) were found for all measured variables, with the exception of the KM score (*F* = 1.773; *p* = 0.156). When comparing participants according to age, significant differences were found in all variables (*F* = 78.554−9.563; *p* <0.001−0.002), except for FM, ∑3-skinfolds, KM score and the distance and the VO2max in the YoYo test (*F* = 3.603−0.266; *p* = 0.060−0.607). The interaction between category and age also showed significant differences in all measured variables (*F* = 43.773–3.196; *p* < 0.001–0.015), except for the KM score (*F* = 1.446; *p* = 0.223).

**Table 1:** Descriptive data (mean ± standard deviation) and differences according to category, including main effects of the covariate age and its intercept.

Variable	Category (Mean ± SD)				Category			Age			Category*age		
	Infant (*n* = 35)	Cadet (*n* = 46)	Junior (*n* = 26)	Senior (*n* = 26)	F	*p*	*η* ^2^_p_	F	*p*	*η* ^2^_p_	F	*p*	*η* ^2^_p_
Body Mass (kg)	52.80 ± 12.15	61.94 ± 12.79	72.69 ± 11.08	77.11 ± 15.78	21.72	**0.000**	0.336	43.721	**0.000**	0.250	15.375	**0.000**	0.325
Height (m)	1.59 ± 0.10	1.72 ± 0.06	1.77 ± 0.06	1.75 ± 0.05	36.81	**0.000**	0.462	37.631	**0.000**	0.223	27.125	**0.000**	0.459
BMI (kg/m2)	20.53 ± 3.68	20.90 ± 3.54	23.05 ± 3.22	24.94 ± 4.24	9.424	**0.000**	0.180	21.958	**0.000**	0.144	6.530	**0.000**	0.169
FM (%)	21.44 ± 9.67	14.52 ± 7.04	15.07 ± 7.01	17.56 ± 9.34	5.250	**0.002**	0.109	1.287	0.259	0.100	4.013	**0.004**	0.111
∑3-skinfolds (mm)	46.79 ± 19.60	32.38 ± 17.88	33.06 ± 16.74	41.18 ± 26.15	4.259	**0.007**	0.090	0.266	0.607	0.002	3.196	**0.015**	0.091
MM (kg)	22.31 ± 4.86	26.74 ± 5.87	32.64 ± 4.79	34.68 ± 6.40	31.706	**0.000**	0.424	68.840	**0.000**	0.344	23.249	**0.000**	0.421
∑3-girths sum corrected (mm)	98.19 ± 10.82	105.21 ± 12.17	115.76 ± 8.97	118.89 ± 13.49	22.990	**0.000**	0.348	49.539	**0.000**	0.274	16.631	**0.000**	0.342
KM	7.40 ± 1.98	6.37 ± 2.21	6.92 ± 2.46	6.38 ± 2.15	1.773	0.156	0.040	0.328	0.568	0.003	1.446	0.223	0.043
MB Throw (m)	4.39 ± 0.99	6.26 ± 1.13	7.66 ± 1.24	7.60 ± 1.14	58.408	**0.000**	0.576	78.554	**0.000**	0.375	43.773	**0.000**	0.578
T-Half Test (s)	7.05 ± 0.71	6.55 ± 0.64	6.01 ± 0.53	6.45 ± 0.47	14.872	**0.000**	0.257	8.480	**0.004**	0.061	11.428	**0.000**	0.263
SJ (cm)	18.94 ± 6.16	25.45 ± 4.72	30.28 ± 4.97	24.82 ± 6.61	21.423	**0.000**	0.333	10.423	**0.002**	0.074	16.254	**0.000**	0.337
CMJ (cm)	20.85 ± 6.26	29.87 ± 5.20	34.77 ± 5.90	30.79 ± 6.43	31.790	**0.000**	0.425	22.293	**0.000**	0.145	23.978	**0.000**	0.428
Sprint (s)	4.76 ± 0.34	4.29 ± 0.32	4.09 ± 0.22	4.31 ± 0.36	25.748	**0.000**	0.375	9.563	**0.002**	0.068	20.999	**0.000**	0.396
YoYo distance (m)	757 ± 453	1142 ± 420	1083 ± 415	1091 ± 452	5.911	**0.001**	0.121	3.603	0.060	0.027	4.517	**0.002**	0.124
YoYo VO_2_ max (mL/min/kg)	42.75 ± 3.80	45.99 ± 3.53	45.50 ± 3.49	45.56 ± 3.79	5.911	**0.001**	0.121	3.603	0.060	0.027	4.517	**0.002**	0.124

**Notes.**

SD standard deviation BMI Body Mass Index FM Fat mass ∑3-skinfolds Sum of 3-skinfolds MM Muscle mass ∑3-girths sum corrected Sum of corrected girths KM Kidmed MB Medicine ball SJ Squad Jump CMJ Counter Movement Jump

Values in bold are statistically significant (*p* < 0.05).

With respect to the anthropometric variables (Table 2), it was found that the infant category showed significantly lower values with the majority of the other categories in body mass, height, BMI, MM and girths corrected sum, and significantly higher values respect the majority of the other categories in the percentage of FM and ∑3-skinfolds (*p* < 0.002–0.021. The cadet category compared to the junior category, showed significantly lower values in body mass, height, MM and ∑3-girths sum corrected (*p* < 0.001–0.009). Regarding the comparison between cadets and seniors, it was found that cadets showed significantly lower values in body mass, BMI, MM and ∑3-girths sum corrected (*p* < 0.001). No significant differences were found between junior and senior categories (*p* > 0.05).

**Table 2:** *Post hoc* comparison between categories with significant differences in ANCOVA analysis for kinanthropometric characteristics.

Variable	Group comparison		Model					
			Category			Category*age		
			Mean difference ± SD	*p*	95% CI	Mean difference ± SD	*p*	95% CI
Body mass (kg)	I	C	−9.14 ± 2.91	**0.012**	−16.93 to −1.34	−8.80 ± 3.17	**0.038**	−17.29 to −0.31
	I	J	−19.89 ± 3.36	**0.000**	−28.88 to −10.89	−18.99 ± 4.71	**0.001**	−31.64 to −6.35
	I	S	−24.31 ± 3.36	**0.000**	−33.30 to −15.31	−22.73 ± 6.72	**0.006**	−40.75 to −4.72
	C	J	−10.75 ± 3.18	**0.006**	−19.27 to −2.22	−10.19 ± 3.80	**0.050**	−20.38 to −0.00
	C	S	−15.17 ± 3.18	**0.000**	−23.69 to −6.65	−13.93 ± 5.58	0.083	−28.89 to 1.029
	J	S	−4.42 ± 3.59	1.000	−14.05 to 5.21	−3.740 ± 4.39	1.000	−15.53 to 8.05
Height (m)	I	C	−0.12 ± 0.02	**0.000**	−0.16 to −0.07	−0.12 ± 0.02	**0.000**	−0.16 to −0.07
	I	J	−0.17 ± 0.02	**0.000**	−0.23 to −0.12	−0.17 ± 0.03	**0.000**	−0.24 to −0.09
	I	S	−0.15 ± 0.02	**0.000**	−0.21 to −0.10	−0.14 ± 0.04	**0.001**	−0.24 to −0.04
	C	J	−0.06 ± 0.018	**0.009**	−0.11 to −0.10	−0.05 ± 0.02	0.079	−0.11 to 0.00
	C	S	−0.04 ± 0.08	0.223	−0.08 to −0.01	−0.03 ± 0.03	1.000	−0.11 to 0.06
	J	S	0.02 ± 0.02	1.000	−0.03 to 0.07	0.03 ± 0.02	1.000	−0.04 to 0.09
BMI (kg/m2)	I	C	−0.37 ± 0.83	1.000	−2.59 to 1.84	−0.29 ± 0.90	1.000	−2.69 to 2.14
	I	J	−2.52 ± 0.95	0.056	−5.08 to 0.04	−2.276 ± 1.34	0.554	−5.873 to 1.32
	I	S	−4.41 ± 0.95	**0.000**	−6.97 to −1.85	−2.276 ± 1.34	0.236	−9.11 to 1.14
	C	J	−2.15 ± 0.90	0.115	−4.57 to 0.28	−1.99 ± 1.08	0.404	−4.89 to 0.90
	C	S	−4.04 ± 0.90	**0.000**	−6.47 to −1.61	−3.70 ± 1.59	0.128	−7.96 to 0.55
	J	S	−1.89 ± 1.02	0.400	−4.63 to 0.85	−1.71 ± 1.25	1.000	−5.01 to 1.65
FM (%)	I	C	6.92 ± 1.85	**0.002**	1.95 to 11.89	6.67 ± 2.02	**0.008**	1.25 to 12.08
	I	J	6.36 ± 2.14	**0.021**	0.63 to 12.10	5.69 ± 3.01	0.363	−2.37 to 13.76
	I	S	3.88 ± 2.14	0.433	−1.85 to 9.62	2.70 ± 4.29	1.000	−8.79 to 14.19
	C	J	−0.55 ± 2.03	1.000	−5.99 to 4.88	−0.93 ± 2.42	1.000	−7.47 to 5.53
	C	S	−3.04 ± 2.03	0.821	−8.47 to 2.40	−3.96 ± 3.56	1.000	−13.51 to 5.58
	J	S	−2.48 ± 2.29	1.000	−8.63 to 3.66	−2.99 ± 2.80	1.000	−10.51 to 4.52
∑3-skinfolds (mm)	I	C	14.51 ± 4.48	**0.009**	2.49 to 26.52	14.31 ± 4.89	**0.024**	1.21 to 27.41
	I	J	13.73 ± 5.17	0.054	−0.14 to 27.59	13.19 ± 7.27	0.433	−6.30 to 32.69
	I	S	5.61 ± 5.17	1.000	−8.25 to 19.48	4.67 ± 10.37	1.000	−23.11 to 32.45
	C	J	−0.78 ± 4.90	1.000	−13.92 to 12.36	−1.12 ± 5.86	1.000	−16.83 to 14.59
	C	S	−8.89 ± 4.90	0.432	−22.03 to 4.25	−9.64 ± 8.61	1.000	−32.71 to 13.43
	J	S	−8.11 ± 5.54	0.875	−22.97 to 6.74	−8.52 ± 6.78	1.000	−26.70 to 9.66
MM (kg)	I	C	−4.43 ± 1.24	**0.003**	−7.76 to −1.01	−3.85 ± 1.35	**0.030**	−7.46 to −0.23
	I	J	−10.33 ± 1.43	**0.000**	−14.17 to −6.49	−8.76 ± 2.01	**0.000**	−14.14 to −3.39
	I	S	−12.37 ± 1.43	**0.000**	−16.21 to −8.53	−9.61 ± 2.86	**0.006**	−17.27 to −1.95
	C	J	−5.90 ± 1.36	**0.000**	−9.54 to −2.26	−4.92 ± 1.62	**0.017**	−9.25 to −0.59
	C	S	−7.93 ± 1.36	**0.000**	−11.58 to −4.30	−5.76 ± 2.37	0.099	−12.12 to 0.59
	J	S	−2.04 ± 1.53	1.000	−6.15 to 2.08	−0.84 ± 1.87	1.000	−5.86 to 4.17
∑3-girths corrected sum (mm)	I	C	−7.01 ± 2.47	**0.032**	−13.63 to −0.39	−6.26 ± 2.69	0.129	−13.47 to 0.95
	I	J	−17.56 ± 2.85	**0.000**	−25.20 to −9.92	−15.55 ± 4.00	**0.001**	−26.27 to −4.83
	I	S	−20.70 ± 2.85	**0.000**	−28.34 to −13.05	−17.15 ± 5.70	**0.019**	−32.44 to −1.87
	C	J	−10.55 ± 2.70	**0.001**	−17.79 to −3.30	−9.29 ± 3.22	**0.028**	−17.93 to −0.65
	C	S	−13.68 ± 2.70	**0.000**	−20.92 to −6.44	−10.89 ± 4.74	0.138	−23.59 to 1.79
	J	S	−3.15 ± 3-05	1.000	−11.32 to 5.05	−1.60 ± 3.73	1.000	−11.60 to 8.39

**Notes.**

SD standard deviation BMI Body Mass Index FM Fat mass ∑3-skinfolds Sum of 3-skinfolds MM Muscle mass ∑3 corrected PR Sum of corrected girths

Values in bold are statistically significant (*p* < 0.05).

Regarding the differences in the physical fitness variables between categories (Table 3), the infant category showed a worse sports performance in the medicine ball throw, T-Half test, SJ, CMJ, sprint and Yo-Yo test variables (*p* < 0.000–0.026). The cadet category compared to the junior category, showed significantly worse performance in the medicine ball throw, T-Half test, SJ y CMJ (*p* < 0.001–0.005). Regarding the comparison between cadets and seniors, it was found that cadets showed significantly lower distance in the medicine ball throw (*p* < 0.001). On the other hand, in the comparison of the junior and senior categories, junior category showed significantly higher differences in the SJ jump test (*p* < 0.003). The category*age group interaction showed that age had a significant influence (*p* < 0.001−0.038) on most of the variables studied as soon as the infant and cadet categories were compared with any of the other categories, with the exception of the BMI variable which showed no differences in any comparison (*p* = 0.236−1.000).

**Table 3:** *Post hoc* comparison between categories with significant differences in the ANCOVA analysis for physical capacity results.

Variable	Group comparison		Model					
			Category			Category*age		
			Mean difference ± SD	*p*	95% CI	Mean difference ± SD	*p*	95% CI
MB Throw (m)	I	C	−1.87 ± 0.25	**0.000**	−2.54 to −1.19	−1.74 ± 0.27	**0.000**	−2.47 to −1.01
	I	J	−3.26 ± 0.29	**0.000**	−4.04 to −2.49	−2.91 ± 0.40	**0.000**	−3.99 to −1.83
	I	S	−3.21 ± 0.29	**0.000**	−3.99 to −2.43	−2.59 ± 0.58	**0.000**	−4.14 to −1.05
	C	J	−1.39 ± 0.27	**0.000**	−2.13 to −0.66	−1.17 ± 0.33	**0.003**	−2.05 to −0.30
	C	S	−1.34 ± 0.27	**0.000**	−2.08 to −0.60	−0.85 ± 0.48	0.462	−2.14 to 0.43
	J	S	0.05 ± 0.31	1.000	−0.78 to 0.88	0.32 ± 0.38	1.000	−0.69 to 1.33
T-Half Test (s)	I	C	0.50 ± 0.14	**0.002**	0.13 to 0.87	0.57 ± 0.15	**0.001**	0.17 to 0.97
	I	J	1.04 ± 0.16	**0.000**	0.62 to 1.47	1.22 ± 0.22	**0.000**	0.63 to 1.81
	I	S	0.60 ± 0.16	**0.001**	0.18 to 1.02	0.91 ± 0.32	**0.026**	0.069 to 1.76
	C	J	0.54 ± 0.15	**0.003**	0.14 to 0.94	0.65 ± 0.18	**0.002**	0.17 to 1.13
	C	S	0.10 ± 0.15	1.000	−0.30 to 0.50	0.35 ± 0.26	1.000	−0.36 to 1.05
	J	S	−0.44 ± 0.17	0.063	−0.89 to 0.01	−0.30 ± 0.21	0.849	−0.86 to 0.25
SJ (cm)	I	C	−6.51 ± 1.25	**0.000**	−9.86 to −3.16	−6.54 ± 1.36	**0.000**	−10.19 to −2.89
	I	J	−11.34 ± 1.44	**0.000**	−15.20 to −7.47	−11.42 ± 2.03	**0.000**	−16.85 to −5.99
	I	S	−5.87 ± 1.44	**0.000**	−9.74 to −2.01	−6.01 ± 2.89	0.236	−13.76 to 1.73
	C	J	−4.83 ± 1.37	**0.003**	−8.49 to −1.18	−4.88 ± 1.63	**0.020**	−9.26 to −0.502
	C	S	0.64 ± 1.37	1.000	−3.02 to 4.29	0.52 ± 2.39	1.000	−5.90 to 6.95
	J	S	5.47 ± 1.54	**0.003**	1.33 to 9.60	5.40 ± 1.89	**0.030**	0.34 to 10.47
CMJ (cm)	I	C	−9.02 ± 1.32	**0.000**	−12.55 to −5.49	−9.04 ± 1.44	**0.000**	−12.89 to −5.19
	I	J	−13.92 ± 1.52	**0.000**	−17.99 to −9.85	−13.98 ± 2.14	**0.000**	−19.71 to −8.25
	I	S	−9.94 ± 1.52	**0.000**	−14.02 to −5.87	−10.04 ± 3.05	**0.008**	−18.21 to −1.87
	C	J	−4.90 ± 1.44	**0.005**	−8.76 to −1.04	−4.94 ± 1.72	**0.029**	−9.56 to −0.32
	C	S	−0.92 ± 1.44	1.000	−4.79 to 2.94	−1.00 ± 2.53	1.000	−7.78 to 5.78
	J	S	3.98 ± 1.63	0.096	−0.39 to 8.34	3.94 ± 1.99	0.303	−1.41 to 9.28
Sprint (s)	I	C	0.48 ± 0.07	**0.000**	0.28 to 0.66	0.53 ± 0.08	**0.000**	0.33 to 0.73
	I	J	0.67 ± 0.08	**0.000**	0.45 to 0.89	0.84 ± 0.11	**0.000**	0.53 to 1.14
	I	S	0.46 ± 0.08	**0.000**	0.24 to 0.68	0.75 ± 0.16	**0.000**	0.32 to 1.18
	C	J	0.20 ± 0.08	0.062	−0.01 to 0.41	0.31 ± 0.09	**0.006**	0.06 to 0.55
	C	S	−0.01 ± 0.08	1.000	−0.22 to 0.19	0.22 ± 0.13	0.134	−0.14 to 0.58
	J	S	−0.21 ± 0.09	0.108	−0.44 to 0.02	−0.08 ± 0.11	1.000	−0.37 to 0.20
YoYo (m)	I	C	−385 ± 97	**0.001**	−646 to −124	−457 ± 128	**0.003**	−802 to −113
	I	J	−326 ± 112	**0.026**	−627 to −25	−394 ± 188	0.228	−898 to 110
	I	S	−334 ± 112	**0.021**	−635 to −33	−404 ± 254	0.680	−1084 to 275
	C	J	59 ± 106	1.000	−227 to 344	63 ± 133	1.000	−294 to 421
	C	S	51 ± 106	1.000	−234 to 336	53 ± 190	1.000	−457 to 563
	J	S	8 ± 120	1.000	−330 to 315	−10 ± 132	1.000	−364 to 344
YoYo VO_2_ max (mL/min/kg)	I	C	−3.23 ± 0.82	**0.001**	−5.43 to −1.04	−3.84 ± 1.08	**0.003**	−6.74 to −0.95
	I	J	−2.74 ± 0.94	**0.026**	−5.27 to −0.21	−3.31 ± 1.58	0.228	−7.54 to 0.92
	I	S	−2.81 ± 0.94	**0.021**	−5.34 to −0.28	−3.40 ± 2.13	0.680	−9.10 to 2.31
	C	J	0.49 ± 0.89	1.000	−1.90 to 2.89	0.53 ± 1.12	1.000	−2.47 to 3.53
	C	S	0.43 ± 0.89	1.000	−1.97 to 2.83	0.45 ± 1.60	1.000	−3.84 to 4.73
	J	S	−0.06 ± 1.01	1.000	−2.77 to 2.65	−0.08 ± 1.11	1.000	−3.06 to 2.89

**Notes.**

SD standard deviation MB Medicine ball SJ Squad Jump CMJ Counter Movement Jump

Values in bold are statistically significant (*p* < 0.05).

Table 4 shows the distribution (%) of the degree of adherence to MD, calculated by the score obtained in the KIDMED questionnaire, according to category. No significant difference was found between categories (*p* = 0.204). However, the infant and senior categories had the fewest players with poor adherence (2.9% and 7.7%, respectively), while the infant and junior categories had the most players with good adherence (54.3% and 50.0%, respectively). With respect to the total, only 10.5% had a poor level of adherence, while the predominant level was medium with 47.4%.

**Table 4:** Distribution (%) of the level of adherence to the Mediterranean diet according to category.

Adhesion	Infant (*n* = 35)	Cadet (*n* = 46)	Junior (*n* = 26)	Senior (*n* = 26)	Total (*n* = 133)	X^2^/*p*
Good (%)	54.3	32.6	50.0	34.6	42.1	X^2^ = 8.501; *p*= 0.204
Average (%)	42.9	52.2	34.6	57.7	47.4	
Poor (%)	2.9	15.2	15.4	7.7	10.5	

**Figure 1:**
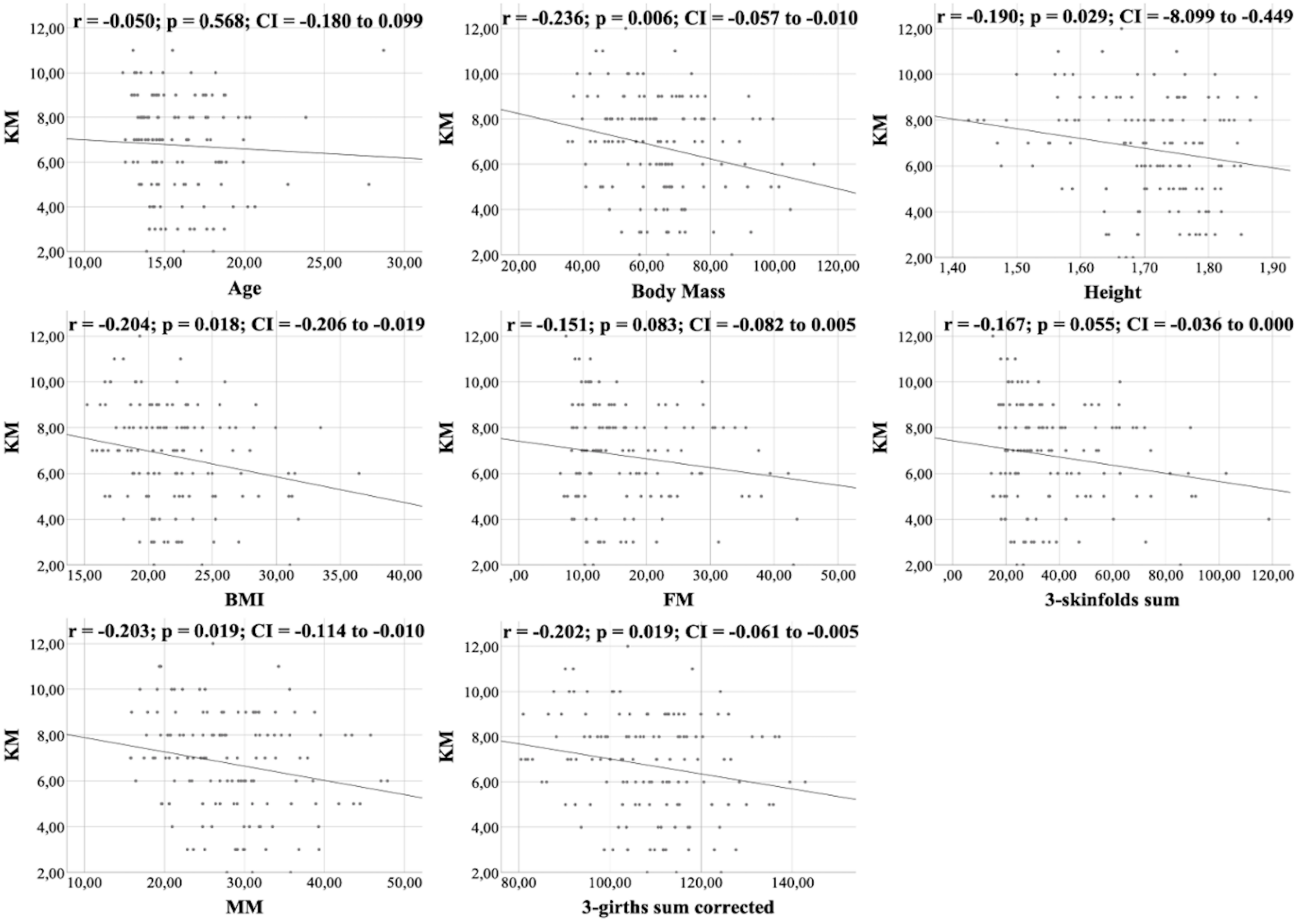
Correlations between KIDMED score and kinanthropometric characteristics.

**Figure 2:**
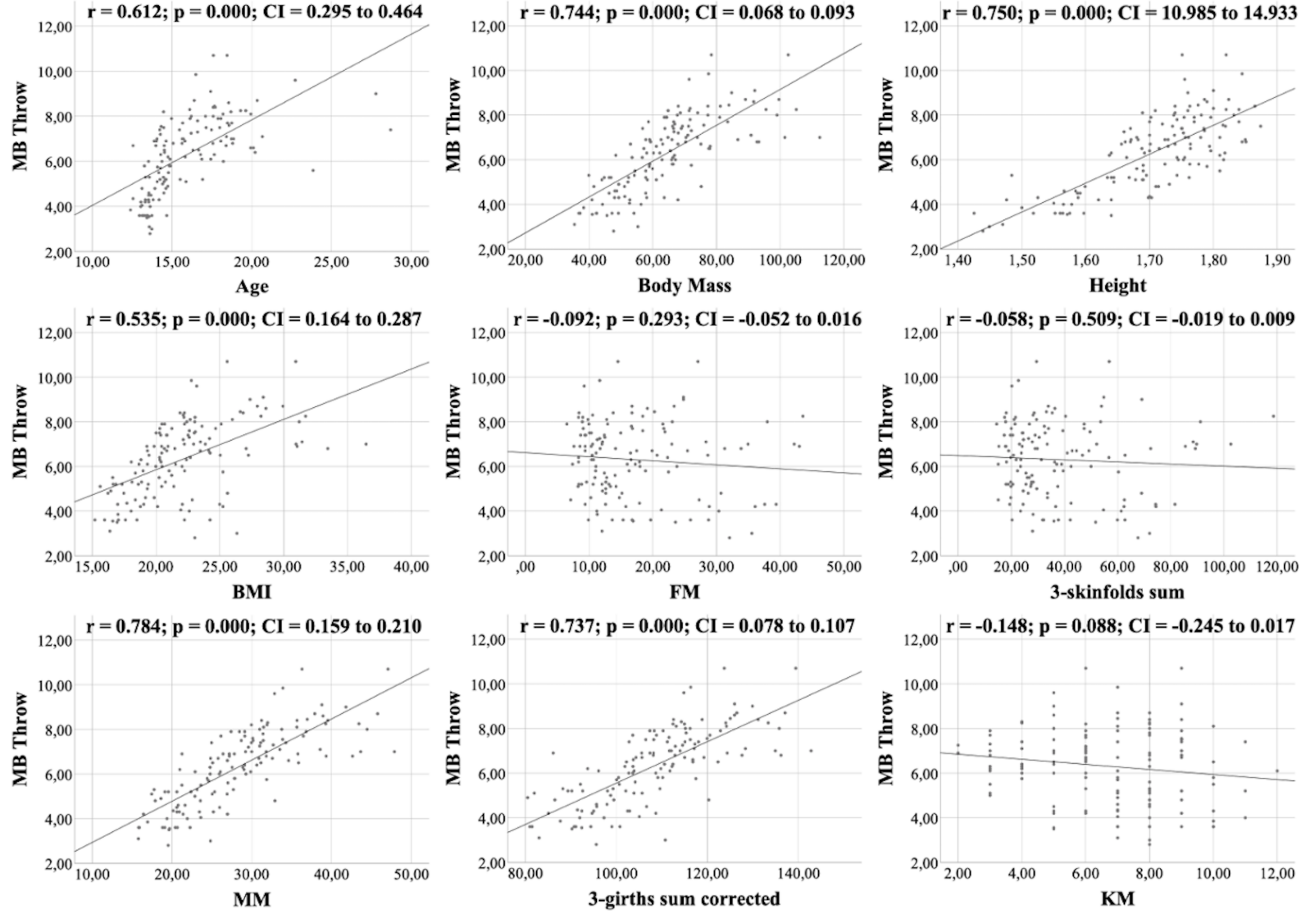
Correlations between medicine ball throwing, kinanthropometric characteristics and KIDMED score.

**Figure 3:**
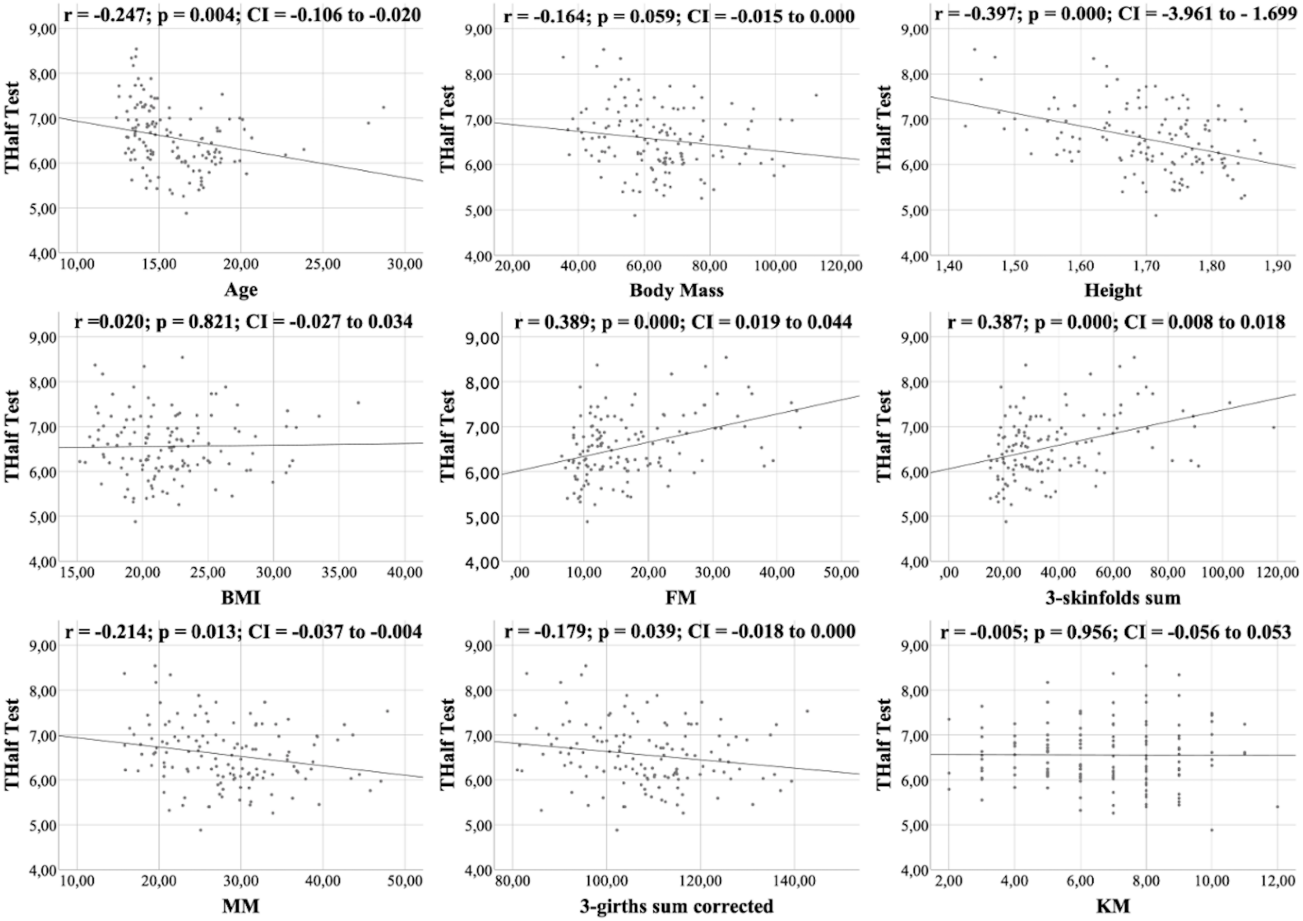
Correlations between T-Half test, kinanthropometric characteristics and KIDMED score.

**Figure 4:**
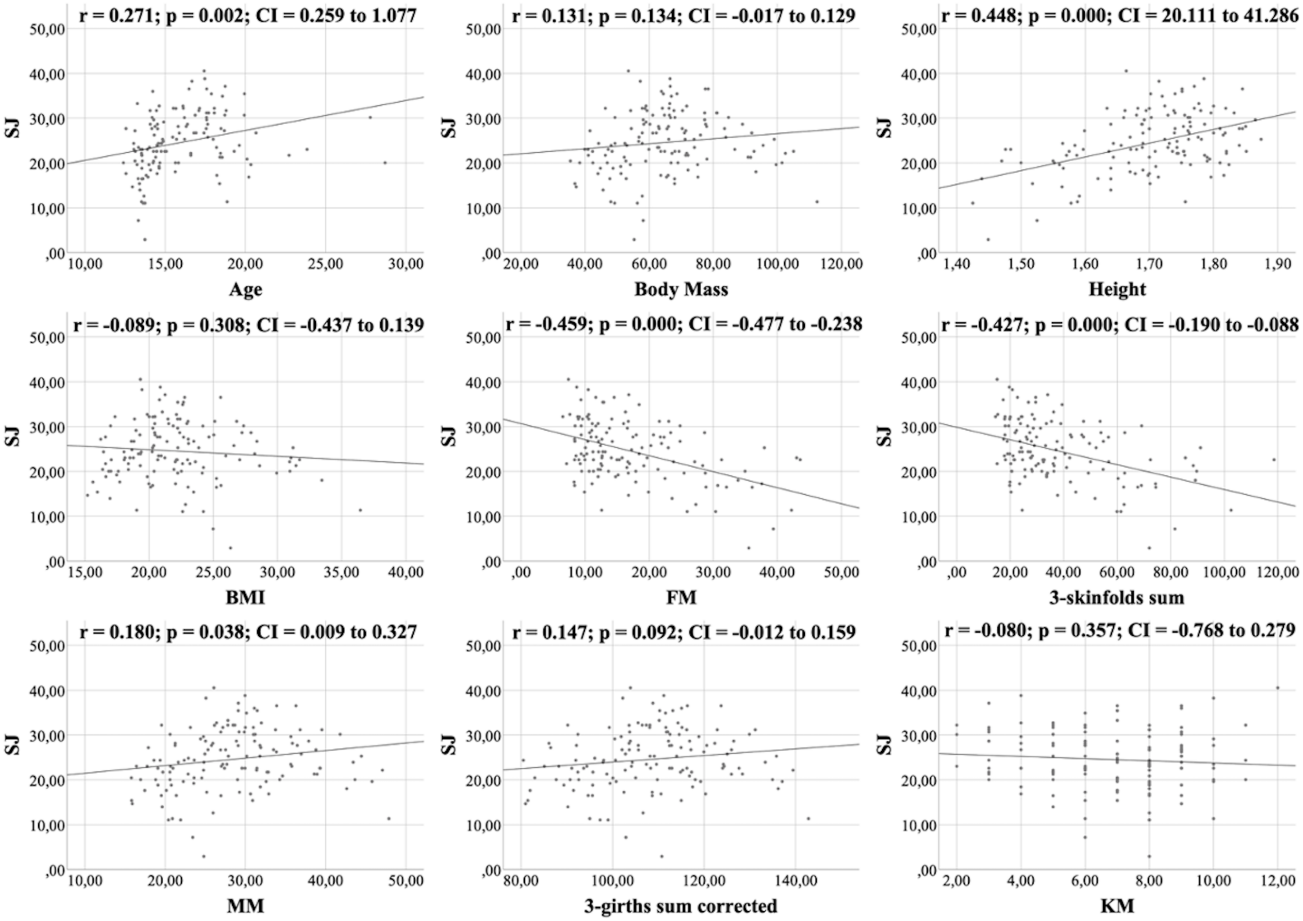
Correlations between the SJ test, kinanthropometric characteristics and the KIDMED score.

**Figure 5:**
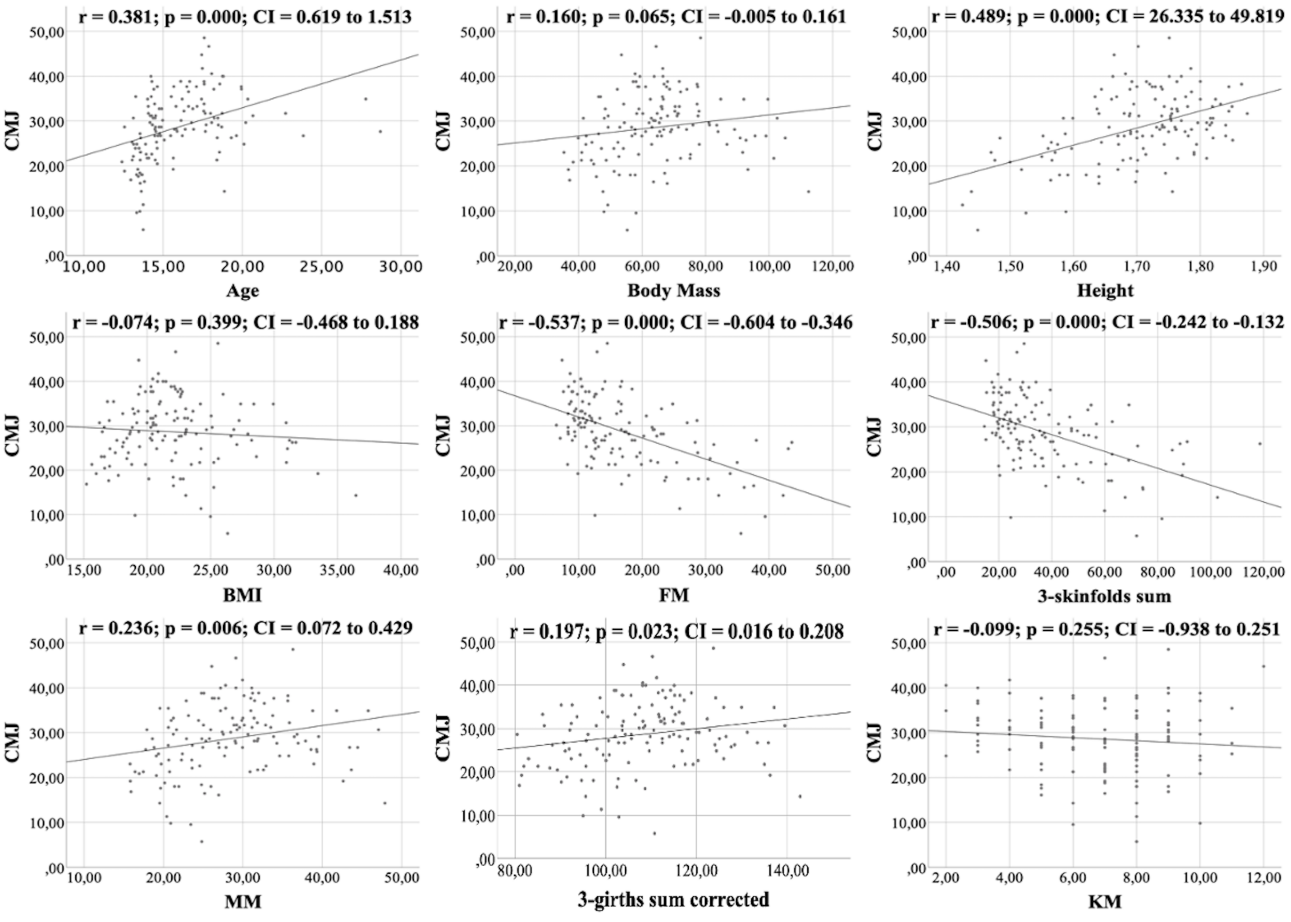
Correlations between the CMJ test, kinanthropometric characteristics and the KIDMED score.

**Figure 6:**
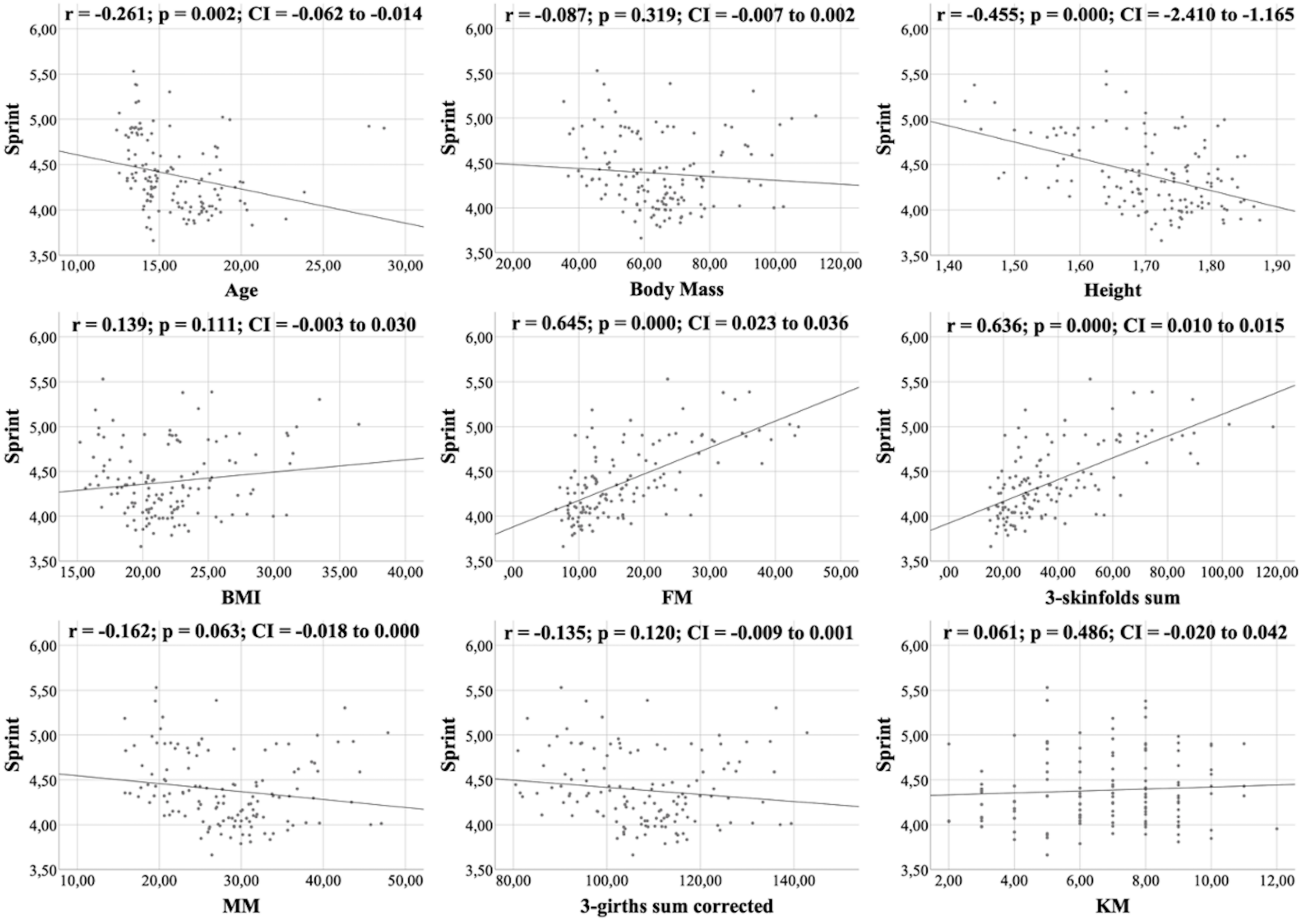
Correlations between sprint, kinanthropometric characteristics and KIDMED score.

**Figure 7:**
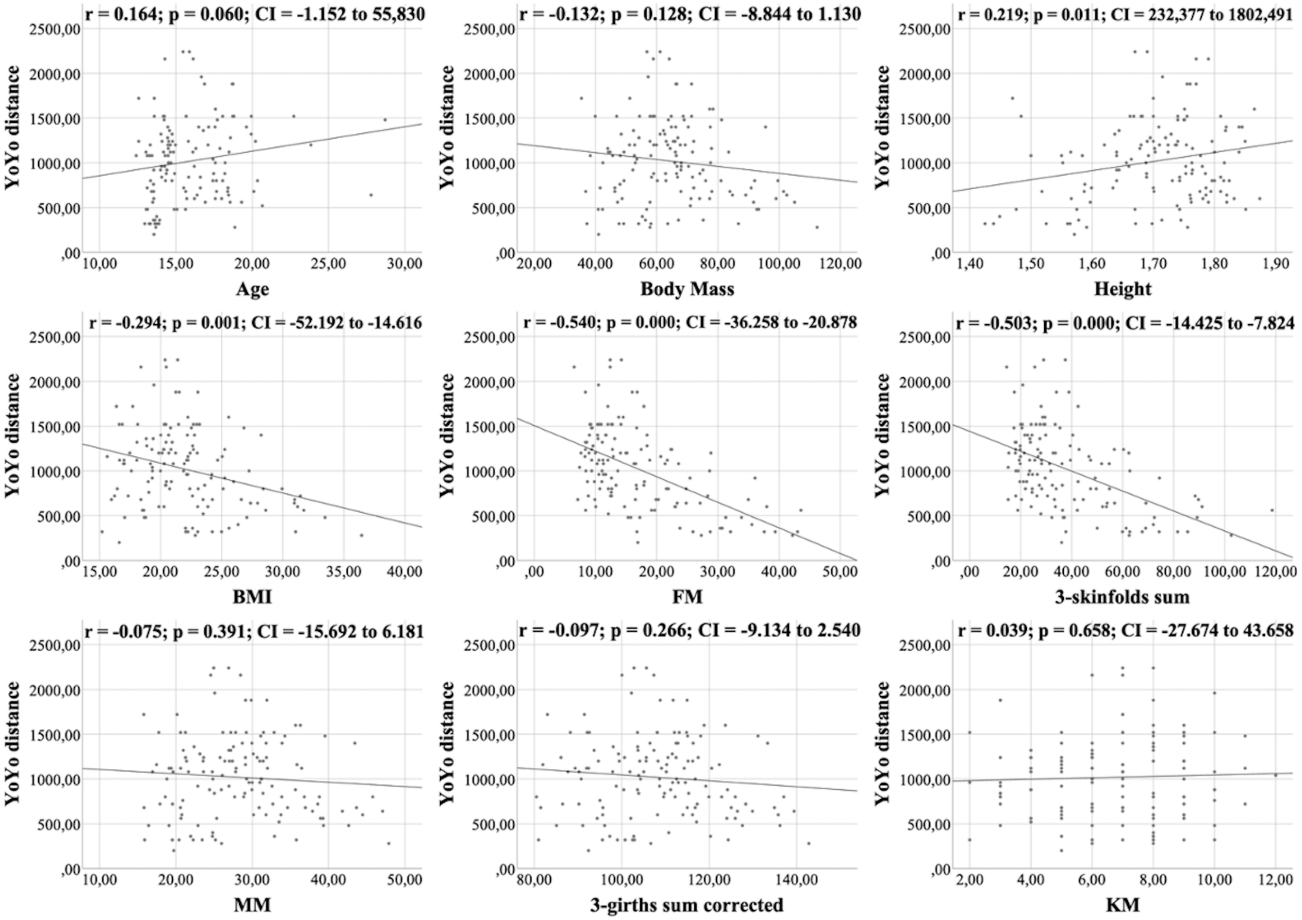
Correlations between YOYO test distance, kinanthropometric characteristics and KIDMED score.

**Figure 8:**
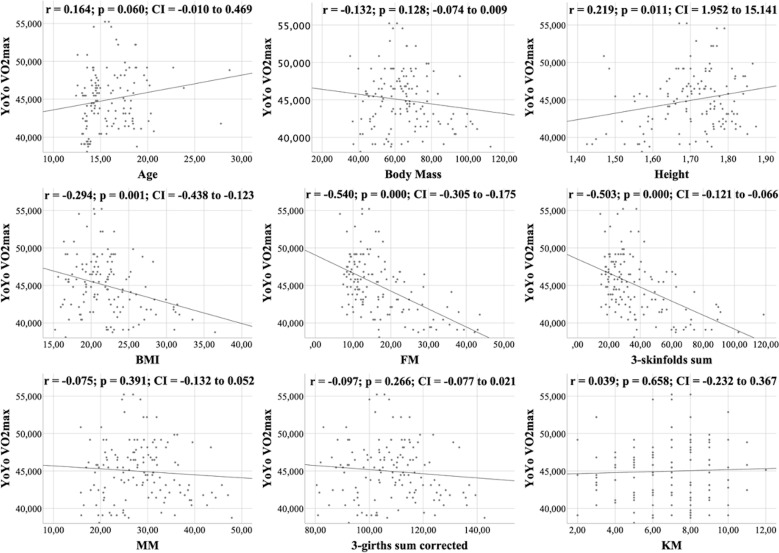
Correlations between YOYO test VO_2_ max, kinanthropometric characteristics and KIDMED score.

All figures show the bivariate correlations between anthropometric characteristics and physical test results, and between KIDMED score and both. The KIDMED questionnaire showed low negative correlations with body mass, height, BMI, MM and ∑3-girths sum corrected (*r* = −0.190 to −0.236; *p* < 0.006−0.019), whereas it did not correlate with any variable from the the physical tests (*r* = −0.148 to 0.061; *p* = 0.088−0.956) (Fig. 1). Upper limb power, assessed with the medicine ball throw, showed high positive correlations with age, body mass, height, BMI, MM and ∑3-girths sum corrected (*r* = 0.535 to 0.784; *p* < 0.001) (Fig. 2). In the agility test (T-Half Test), low to moderate negative correlations were found between the time taken to complete the run, with age, height, MM and ∑3-girths sum corrected (*r* = −0.397 to −0.179; *p* < 0.001−0.039), and moderate positive correlations with both FM and ∑3-skinfolds (*r* = 0.387 to 0.389; *p* < 0.001−0.03) (Fig. 3). The SJ (Fig. 4) and CMJ (Fig. 5) showed low to moderate positive correlations with age, height, and MM (*r* = 0.180 to 0.489; *p* < 0.001−0.038), and low to moderate negative correlations with FM and the ∑3-skinfolds (*r* = −0.427 to −0.537; *p* < 0.001). In addition, the CMJ was also positively correlated with a low association with the ∑3-girths sum corrected (*r* = 0.197; *p* < 0.023). In speed, with a longer time in sprint indicating a worse performance, low to moderate negative correlations were observed with both age and height (*r* = −0.261 to −0.445; *p* < 0.001–0.002), and high positive correlations with both FM and ∑3-skinfolds (*r* = 0.636 to 0.645; *p* < 0.001) (Fig. 6). Regarding endurance, both distance (Fig. 7) and VO_2_ max (Fig. 8), low to high negative correlations were observed with BMI, FM and ∑3-skinfolds (*r* = −0.540 to −0.294; *p* < 0.001), and low positive correlation with height (*r* = 0.219; *p* < 0.011).

Table 5 shows the multiple linear regression analysis, as well as the resulting predictive equations for physical capacity and KIDMED score. With the exception of the latter, where the independent variable included only explained 7% of its variability (*p* = 0.006), the anthropometric characteristics included in each model explained 32% to 72% of the variability of the physical test results (*p* < 0.001). The most determinant anthropometric characteristics were MM and FM.

**Table 5:** Analysis and regression models of performance in the different physical capacities.

Variable	Analysis	R^2^	*p*	Independent variables included	Standard coefficient (ß)	Equation
KIDMED	Model 1	0.07	**0.006**	Body Mass	−0.24	KIDMED = 8.90 − 0.03*Body Mass
MB Throw	Model 1	0.61	**0.000**	MM	0.78	MB Throw = 1.09 + 0.18*MM
	Model 2	0.72	**0.000**	MM	1.37	MB Throw = 3.40 + 0.32*MM - 0.28*BMI
				BMI	−0.67	
T-Half Test	Model 1	0.16	**0.000**	Height	−0.40	T-Half Test = 11.38 - 2.83*Height
	Model 2	0.29	**0.000**	Height	−0.38	T-Half Test = 8.67 - 2.69*Height + 0.01* ∑3-skinfolds
				∑3-skinfolds	0.37	
	Model 3	0.33	**0.000**	Height	−0.15	T-Half Test = 7.06 - 1.10*Height + 0.02* ∑3-skinfolds - 0.03*MM
				∑3-skinfolds	0.51	
				MM	−0.32	
SJ	Model 1	0.21	**0.000**	FM	−0.46	SJ = 30.65 - 0.36*FM
	Model 2	0.38	**0.000**	FM	−0.42	SJ = -17.83 - 0.33*FM + 28.16*Height
				Height	0.41	
CMJ	Model 1	0.29	**0.000**	FM	−0.54	CMJ = 36.74 - 0.47*FM
	Model 2	0.51	**0.000**	FM	−0.72	CMJ = 24.42 - 0.64*FM + 0.53*MM
				MM	0.50	
Sprint	Model 1	0.42	**0.000**	FM	0.64	Sprint = 3.88 + 0.03*FM
	Model 2	0.57	**0.000**	FM	0.61	Sprint = 6.59 + 0.03*FM - 1.58*Height
			**0.000**	Height	−0.39	
YoYo distance	Model 1	0.29	**0.000**	FM	−0.54	YoYo distance = 1505 - 28.57*FM
	Model 2	0.32	**0.000**	FM	−0.52	YoYo distance = 123 - 27.77*FM + 803*Height
				Height	0.17	
YoYo VO_2_ max	Model 1	0.29	**0.000**	FM	−0.54	YoYo VO_2_ max = 49.05 –0.24*FM
	Model 2	0.32	**0.000**	FM	−0.52	YoYo VO_2_ max = 37.43 –0.23*FM + 6.75*Height
				Height	0.17	

**Notes.**

BMI Body Mass Index FM Fat mass ∑3-skinfolds sum of 3-skinfolds MM Muscle mass MB Medicine ball SJ Squad Jump CMJ Counter Movement Jump

Values in bold are statistically significant (*p* < 0.05).

## Discussion

The main objective was to observe if there were any differences in the anthropometric characteristics, adherence to MD and physical condition as a function of the sports category in handball. It was found that there were significant differences between the categories compared, both in most of the anthropometric characteristics, and in the results of the physical tests; while no significant differences were found between the groups studied in the distribution of the degree of adherence to the MD. In this study, it was found that when the players were part of a category whose age range was older, most of the anthropometric characteristics also increased, with the exception of FM and ∑3-skinfolds, and that the results in the physical tests were better. Hermassi et al. (2020) also found differences in all kinanthropometric parameters between age groups in handball players, with the exception of body fat percentage. However, for the physical tests, they found no significant differences, with the exception of upper limb power in the medicine ball throw test. This could possibly be due to the fact that their study population were young players aged 10 to 12 years old, so the maturation process, with all the hormonal changes that it entails, had not yet begun. Molina-López et al. (2020) found the same results as the present work for both anthropometric characteristics and physical test results in handball players. Moreover, as in this study, the differences between categories were reduced or even not observed when comparing groups further away from puberty, which could be due to the stabilization of the maximum growth peak, and the end of the maturation process (Albaladejo-Saura et al., 2021). Therefore, as players grow older, their body composition changes (Albaladejo-Saura et al., 2022), as can be hypothesized by analyzing the results of the present investigation, allowing in most cases, to have a better performance in physical fitness tests (Albaladejo-Saura et al., 2021). However, this improvement in body composition and performance is also influenced by other reasons, such as the level of play or hours of training (Hammami et al., 2019), so more research is still lacking for a better understanding of the factors that affect body composition and performance of players.

Another objective of this research was to determine the degree of adherence to the MD of handball players. In this line, the findings that were found indicated that the athletes have a medium and good level of adherence to the MD (47.4% and 42.1%, respectively). These findings are similar to those found in several studies, where it was also described that the predominant degrees of adherence were medium or good (Vaquero-Cristóbal et al., 2018; Manzano-Carrasco et al., 2020a; Martínez-Rodríguez et al., 2021). Along these lines, (Manzano-Carrasco et al., 2020a) evaluated athletes of both sexes, and found that 57.6% of men and 59.8% of women had a medium level of adherence. (Martínez-Rodríguez et al., 2021), who evaluated both male and female beach handball players, found that 66.0% of men and 76.0% of women also had a medium level of adherence. Vaquero-Cristóbal et al. (2018), evaluated elite cadet male canoeists, and found that more than half of the sample had a good degree of adherence to the MD. All of these results are similar to those found in the AdolesHealth study (Galan-Lopez et al., 2019), which evaluated 1,717 adolescents of both sexes, from different European cities and schools, and which found that 54.92% had an average adherence to the Mediterranean diet. However, that the dietary pattern of young athletes is the same as the general adolescent population does not mean that it is optimal, as athletes have different requirements to maintain performance during the competitive season (Thomas, Erdman & Burke, 2016). This is an important point for future research.

On the other hand, and despite the fact that in our sample the predominant levels of adherence were good and medium, no significant differences were found in the distribution of the level of MD adherence. Manzano-Carrasco et al. (2020b), after evaluating young soccer players, also found no significant differences in the KIDMED questionnaire score. This could be due to the fact that the MD is being substituted by foods characteristic of other cultures, mainly affecting adolescents (Rubio-Árias et al., 2015). However, there is a lack of more research that delves deeper into this. Another aspect to highlight is that the degree of adherence to the MD has not been correlated with the results of physical tests, but with some anthropometric characteristics. Vaquero-Cristóbal et al. (2018) found no relationship between adherence to the MD and anthropometric parameters of male canoeists. Martínez-Rodríguez et al. (2021) concluded that having a good MD adherence is not enough for obtaining better results in the performance tests in male and female beach handball players. It has been suggested that this could be due to the homogeneity in the adherence to the MD shown by athletes (Vaquero-Cristóbal et al., 2018). Even then, more research is still lacking, both in handball and in other sports, for an in-depth analysis of a sufficient sample size, of whether having a good adherence to the MD or specific eating habits influences performance and body composition or anthropometric characteristics.

The last objective of this research was to know if there was a relationship between anthropometric characteristics and sports performance. Along this line, the findings indicate that both FM percentage and MM are important variables that describe the variability of the results of the physical tests. More specifically, a low percentage of FM, a good development of MM, or both, improve the results of the physical tests. The findings found regarding FM are in agreement with those found by Hermassi et al. (2021), who evaluated obese and non-obese young male handball players, and concluded that the percentage of FM negatively affected speed, vertical jump and aerobic capacity, three basic and necessary skills for a handball player. In another study by Hermassi, Laudner & Schwesig (2019) conducted with young male handball players, the authors found that, with the exception of speed, there was a negative association between FM and the vertical jump, SJ and CMJ performance tests. However, in another study by Hammami et al. (2019) they found that FM was not related to performance measurements, with the exception of vertical and horizontal jumps. Moncef et al. (2012) also found no correlations between body fat and the vertical jump, SJ and CMJ tests, nor with the endurance capacity of the players. Molina-López et al. (2020), after evaluating young handball players, concluded that MM analysis proved to be more useful than FM assessment in determining performance. These differences could be due to the heterogeneity in the competitive level of the handball players included in the studies, so future lines of research should analyze the specific weight of FM and MM according to the level of the players.

Regarding the limitations of this study, the main one was the sample size. It should also be taken into account that the KIDMED questionnaire does not report the amount of food ingested. Furthermore, as the study was cross-sectional, no MD-based nutritional interventions, which could have improved body composition and performance, could have been performed. As for future research, this study should be replicated with women. Another interesting aspect would be to be able to perform a nutritional intervention based on the MD and to evaluate whether players improve their body composition and performance. In this way, it would be possible to analyze in depth, whether having an excellent association with the MD is related to better performance.

As possible practical applications both for handball professionals (sports federations, clubs, coaches, physical trainers, *etc*.) and for the players themselves, it should be noted that this study shows that the plastic components of body composition (adiposity and muscle development) influence the physical condition tests related to handball performance, so that performance in this sport could be improved by modifying these components. In this way, the anthropometric characteristics of the players should be monitored throughout the season in order to be able to individualize the nutritional and training plans to the needs of each player in the search for the optimization of sports performance (Martínez-Rodríguez et al., 2020; Ghobadi et al., 2013). Another practical application of the present study is that taller players have a competitive advantage in physical fitness tests related to handball performance. Because of the influence of maturation on this parameter (Albaladejo-Saura et al., 2021), the selection of players with early maturation during these ages could bring better short-term performance (Albaladejo-Saura et al., 2022), although it is not clear that this is an advantage when the maturation process is completed and the characteristics are equalized (Albaladejo-Saura et al., 2021). On the other hand, there is no great awareness on the part of the players of the importance of having a healthy diet in the search for the optimization of their sports performance, especially in the pubertal stage, so it would be necessary to carry out informative programs from an early age to increase awareness among athletes on this issue.

## Conclusions

In conclusion, there are differences according to category in most of the kinanthropometric and physical condition variables, but not in the level of adherence to the MD, highlighting that as the players were from a category with an older age range, the anthropometric characteristics and the results of the physical tests were better. In addition, most of the subjects had a medium and good level of adherence to the MD, and very few players showed a poor adherence. However, this level of adherence was not associated to the results of the physical tests. Nevertheless, a relationship between anthropometric characteristics and sports performance was observed; having a low percentage of FM, having a good development of MM, or both, improves the results of the physical tests.

##  Supplemental Information

10.7717/peerj.14329/supp-1 Supplemental Information 1 Raw data

## Additional Information and Declarations

### Data Availability

The following information was supplied regarding data availability: The raw data is available in the Supplementary File.
